# Detection of N-glycolyl-neuraminic acid-containing glycolipids in human skin

**DOI:** 10.3389/fimmu.2023.1291292

**Published:** 2023-11-29

**Authors:** Michela Manni, Natalia Rodrigues Mantuano, Andreas Zingg, Elisabeth A. Kappos, Anna-Janina Behrens, Jonathan Back, Rainer Follador, Amir Faridmoayer, Heinz Läubli

**Affiliations:** ^1^ Department of Biomedicine, University Hospital and University of Basel, Basel, Switzerland; ^2^ Glycoera AG, Wädenswil, Switzerland; ^3^ Department of Plastic, Reconstructive, Aesthetic and Handsurgery, University Hospital and University of Basel, Basel, Switzerland; ^4^ Division of Oncology, University Hospital Basel, Basel, Switzerland

**Keywords:** sialic acid, glycolipids, N-acetyl-neuraminic acid, N-glycolyl-neuraminic acid, cancer

## Abstract

Humans lack the enzyme that produces the sialic acid N-glycolyl neuraminic acid (Neu5Gc), but several lines of evidence have shown that Neu5Gc can be taken up by mammalian food sources and replace the common human sialic acid N-acetyl neuraminic acid (Neu5Ac) in glycans. Cancer tissue has been shown to have increased the presence of Neu5Gc and Neu5Gc-containing glycolipids such as the ganglioside GM3, which have been proposed as tumor-specific antigens for antibody treatment. Here, we show that a previously described antibody against Neu5Gc-GM3 is binding to Neu5GC-containing gangliosides and is strongly staining different cancer tissues. However, we also found a strong intracellular staining of keratinocytes of healthy skin. We confirmed this staining on freshly isolated keratinocytes by flow cytometry and detected Neu5Gc by mass spectrometry. This finding implicates that non-human Neu5Gc can be incorporated into gangliosides in human skin, and this should be taken into consideration when targeting Neu5Gc-containing gangliosides for cancer immunotherapy.

## Introduction

Cancer-associated glycan changes include the truncation of carbohydrate chains, increased N-glycan branching, and changes in sialic acid metabolism such as increased terminal sialic acid concentrations (1, 2). Such cancer-associated glycan changes have been targeted using tumor-specific antibodies to the cancer microenvironment (3, 4). One prominent example is the sialyl-Thomsen nouveau (sTn) antigen, a sialylated-truncated O-glycan (5). A trial targeting this glycan antigen in a phase I trial in solid cancer patients with an antibody–drug conjugate is ongoing (e.g., Seagen Inc, NCT04665921).

Sialic acids are monosaccharides that are commonly found on the outer terminal units of cell surfaces in vertebrates (1, 6). They play a crucial role in the interactions between cells and their microenvironment. The two main types of sialic acids found on mammalian cells are N-glycolyl-neuraminic acid (Neu5Gc) and N-acetyl-neuraminic acid (Neu5Ac) (1, 6, 7). Neu5Gc is particularly notable because it is deficient in humans due to a specific genetic deletion in the CMAH gene, which encodes the hydroxylase responsible for converting CMP-Neu5Ac to CMP-Neu5Gc (7). However, despite this deficiency, Neu5Gc can still be absorbed into human tissues through dietary sources, especially red meat, leading to higher concentrations of Neu5Gc in certain types of cancer (8–12). Breast carcinomas showed anti-Neu5GC reactivity in the majority of tumor cells and some related blood vessels (9). In addition, non-human Neu5Gc has also been detected in other healthy organs and biotherapeutic agents, although usually at rather lower levels in healthy tissue (13–15).

Previously, limited expression of N-glycolyl GM3 (NeuGcGM3) ganglioside in human normal tissues was reported, as well as its presence in melanoma and breast carcinoma using the 14F7 antibody (anti-NeuGcGM3)(16, 17). The expression of NeuGc-GM3 using the same antibody was found in a high proportion of neuroectodermal and Wilms tumors, common pediatric cancers (18, 19). The ganglioside Neu5Gc-GM3 has been previously defined as a potential tumor-specific target with little to no expression in healthy tissue (20–22). We further wanted to define the expression of NeuGc-GM3 ganglioside as a potential tumor target in different malignancies. We therefore performed staining with an 14F7 antibody clone.

## Materials and methods

### Tissue microarray immunohistochemistry

Immunohistochemistry was performed by TriStar Technology Group using a Ventana BenchMark XT staining system. In brief, 4-μm FFPE TMAs (TriStar Technology Group) were deparaffinized and hydrated before antigen retrieval in Tris–EDTA buffer, pH 8.4, 100°C, for 8 min. Slides were then blocked in 3% hydrogen peroxide for 4 min at 37°C. Slides were then incubated with 14F7 (1:8,000 dilution, 1 μg/ml) or control mouse IgG antibodies at 37°C for 12 min. Primary antibodies were detected using the OptiView Universal DAB kit (Ventana, #760-700). TMAs (TA3162, TA2998, TA2472, TA2287, and TA2942) were scored by a trained pathologist at TriStar Technology Group. The score 0 to 3+ measures the intensity of the staining in the sample analyzed, assigning 0 for no staining, 1+ for weak staining, 2+ for intermediate staining, and 3+ for strong staining. The H-score was used to evaluate the degree immunoreactivity. The H-score was calculated using the following formula: three times the percentage of strongly stained cells plus two times the percentage of moderately stained cells, in addition to the percentage of weakly stained cells. This computation yields a numerical range spanning from 0 to 300.

### Fresh human skin preparation

Patients undergoing breast and abdominal reduction were consented and enrolled at the University Hospital of Basel. Fresh human skin was collected in PBS, subcutaneous fat was removed, and skin was extensively cut with a scalpel in a petri dish. Skin was then transferred into a 15-ml tube with 1 ml of the following mix per four punch biopsies: collagenase II (2.5 mg/ml) + collagenase IV (2.5 mg/ml) + DNAse (0.5 mg/ml) in PBS. Skin was then digested for 2 h at 37°C with shaking. Cells were next filtered on a 100-μm filter and washed with 5 ml DMEM+10% FBS. The cells were resuspended in FACS Buffer for staining.

### 14F7 antibody

A 14F7 sequence was obtained from published data (23, 24).

Heavy chain: MERHWIFLFLFSVTAGVHSQVQLQQSGNELAKPGASMKMSCRASGYSFTSYWIHWLKQRPDQGLEWIGYIDPATAYTESNQKFKDKAILTADRSSNTAFMYLNSLTSEDSAVYYCARESPRLRRGIYYYAMDYWGQGTTVTVSS.

Light chain: MVSHLKILGLLLFWISASRGDLVLTQSPATLSVTPGDSVSFSCRASQSISNNLHWYQQRTHESPRLLIKYASQSISGIPSRFSGSGSGTDFTLSIISVETEDFGMYFCQQSNRWPLTFGAGTKLELKRA

Expression and purification of the monoclonal antibody was performed at Evitria AG, Switzerland. In brief, the corresponding cDNA was cloned into the vector system. The vector plasmids were gene synthesized. Plasmid DNA was prepared under low-endotoxin conditions. The DNA concentration was determined by measuring the absorption at a wavelength of 260 nm. Correctness of the sequences was verified with Sanger sequencing.

CHO K1 cells were used for production. Cells were transfected in Evitria’s proprietary transfection reagent, and cells were grown after transfection in an animal-component free, serum-free medium. Supernatant was harvested by centrifugation and subsequent filtration (0.2-μm filter). The antibody was purified using MabSelect™ SuRe™.

### Neu5Gc detection in keratinocytes via HILIC-UPLC FLR/MS

The cell surface of human keratinocytes was treated with endoglycoceramidase I (EGCase I; New England Biolabs) according to the manufacturer’s instructions. In brief, cell pellets were digested with EGCase I for 3 h at 37°C, and the supernatant was collected and dried in a vacuum dryer. Oligosaccharides were thus released *via* the hydrolysis of the β-glycosidic linkage between the oligosaccharide and the ceramide in GM3-type gangliosides. The released oligosaccharides were further fluorescently labeled and analyzed by HILIC-UPLC FLR/MS, as described previously (25). Neu5Ac-GM3 and Neu5Gc-GM3 chemically synthesized standards (from ChemBind LLC) were analyzed as controls alongside the keratinocytes.

### Flow cytometry

Anti-Neu5Gc-ganglioside clone 14F7 was obtained from Evitria AG. Single-cell suspension cells were first stained with Fixable Viability Dye (eBioscience) for 20 min in PBS at room temperature. Cells were washed and then incubated with antibodies (5 μg/ml 14F7) in FACS buffer (PBS + 1% FBS) in the presence of an Fc receptor binding inhibitor (Invitrogen) for 30 min on ice. Cells were washed and primary antibodies detected by staining with a goat anti-mouse IgG conjugated to Alexa Fluor 488 (Jackson ImmunoResearch). Data were acquired using a FACS Fortessa (Beckton Dickinson) and analyzed using FlowJo (TriStar).

### Statistical analysis

In order to test statistical relevant differences, we used non-parametric Mann–Whitney test. We used GraphPad Prism 9.0 for the calculation of statistical differences.

## Results

### Expression of Neu5Gc gangliosides in different human cancers

**Figure 1:**
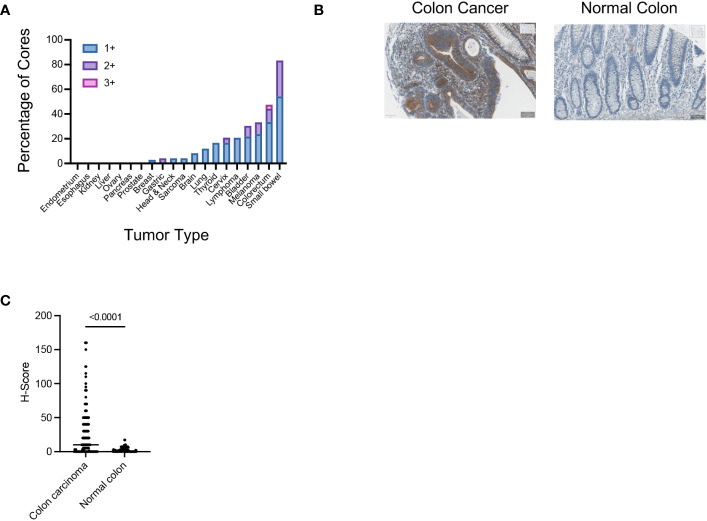
**(A)** Assessment of the Neu5Gc-GM3 expression in the indicated human tumor samples using the 14F7 antibody. Graph shows the percentage of TMA cores which show an IHC score of 1+, 2+, or 3+ in the indicated tumor samples. **(B)** Representative IHC image showing the expression of Neu5Gc-GM3 in colon cancer or normal colon as assessed by 14F7 staining. **(C)** Graph showing the Neu5Gc-GM3 level in colon cancer (140 cores) or normal colon (42 cores) expressed as H-Score. Each dot represents one TMA core.

The 14F7 antibody clone has been previously used to study the expression of Neu5Gc-GM3 in human tissues (22, 26–28). Different cancer types were found to have a high expression of Neu5Gc-GM3, whereas most of the healthy tissue had minimal staining as determined by immunohistochemistry (IHC). We wanted to characterize the binding of the 14F7 antibody to different glycolipids printed on a glass slide. Binding was highly specific to Neu5Gc-containing glycolipids with the strongest binding to Neu5Gc-containing GD3. Binding to Neu5Gc-GM3 was also clearly detectable (**Supplementary Figure S1A**). We next tested the specificity of the 14F7 antibody in an ELISA assay using Neu5Gc-GM3 and Neu5Ac-GM3, which demonstrated specific binding of 14F7 to Neu5Gc-containing gangliosides (**Supplementary Figure S1B**). We used the 14F7 antibody to stain various cancer tissues and analyzed the expression by classifying the staining intensity 1+ to 3+. We found that a significant percentage of TMA cores of intestinal epithelia had a positive staining (**Figure 1A**; **Supplementary Figures S1C, D**; **Table 1**). Several cores of colorectal cancer showed a strong staining (**Figures 1A, B**). When we compared the staining of normal colon tissue with colorectal cancer, we observed a clear increased H-score in many colorectal samples and no or minimal staining in samples of healthy colon tissue (**Figure 1C**).

**Table 1:** Summary of 14F7 IHC results in various tumor tissues.

	No. of cores*					H-score		
Type	0	1+	2+	3+	TOTAL	MIN	MAX	MEAN
Bladder	16	5	2	0	23	0	80	12.8
Brain	22	2	0	0	24	0	17	1.1
Breast	66	2	0	0	68	0	30	0.8
Cervix	19	4	1	0	24	0	60	5.5
Colorectum	97	62	20	6	185	0	160	21.8
Endometrium	24	0	0	0	24	0	2	0.1
Esophagus	24	0	0	0	24	0	5	0.4
Gastric	23	0	1	0	24	0	50	2.1
Head and neck	23	1	0	0	24	0	10	1.1
Kidney	24	0	0	0	24	0	0	0.0
Liver	24	0	0	0	24	0	0	0.0
Lung	132	18	0	0	150	0	30	2.3
Lymphoma	19	5	0	0	24	0	60	5.8
Melanoma	14	5	2	0	21	0	60	10.0
Ovary	24	0	0	0	24	0	5	0.2
Pancreas	22	0	0	0	22	0	5	0.3
Prostate	23	0	0	0	23	0	1	0.0
Sarcoma	23	1	0	0	24	0	60	4.0
Small bowel	4	13	7	0	24	0	110	34.2
Thyroid	20	4	0	0	24	0	20	3.0

Different cancer tissues were stained with the 14F7 antibody, and immunoreactivity was determined by the H-score. The number of cores with different levels of positivity are also shown.

*: Some cores are duplicated, so that the number of cases are less than the number of cores.

Taken together, these data reproduce previous observations showing an enriched staining with the 14F7 antibody in various cancer types and minimal staining in healthy tissue.

### Staining with a Neu5Gc-ganglioside-specific antibody in melanoma and healthy skin

**Figure 2:**
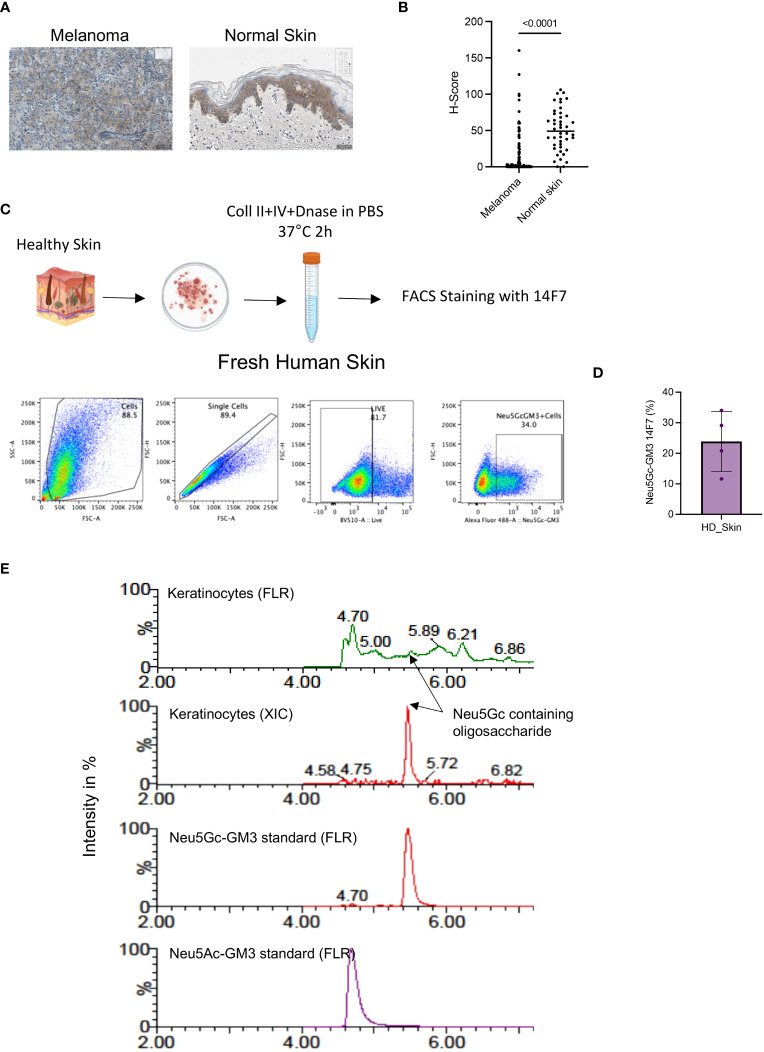
**(A)** Representative IHC image showing the expression of Neu5Gc-GM3 in melanoma or normal skin as assessed by 14F7 staining. **(B)** Quantification of Neu5Gc-GM3 expression as assessed by 14F7 staining. Graph shows the H-score in melanoma (119 cores) and normal skin (44 cores). Each dot represents one TMA core. **(C)** Gating strategy used to evaluate Neu5Gc-GM3 expression in fresh human skin by flow cytometry. **(D)** Quantification of **(C)**. Graph showing the percentage of skin cells expressing Neu5Gc-GM3. Each dot represents a healthy skin donor. **(E)**, HILIC-UPLC-FLR/MS analysis of GM3 in keratinocytes in comparison with a chemically synthesized Neu5Gc-GM3 standard. In the fluorescence trace (FLR), a distinctive peak at the retention time of the Neu5Gc-GM3 control is clearly visible that is confirmed by an extracted ion chromatogram (XIC).

Our analysis also showed a significant staining of melanoma samples (**Figures 2A, B**). However, when we further compared the H-score from a TMA with additional healthy skin samples than used in the initial screening (**Supplementary Figure S1C**), we found a very strong staining of 14F7 which was mainly localized intracellularly in keratinocytes (**Figures 2A, B**). Indeed, the staining of healthy skin was even higher than in melanoma samples as measured by H-score determination (**Figure 2B**).

These data show that the Neu5Gc-specific antibody 14F7 is strongly staining keratinocytes in healthy skin, although it is not excluded that other cell types in skin are also positive for Neu5Gc-containing gangliosides.

### Detection Neu5Gc in human skin

We further wanted to confirm the presence of Neu5Gc in human skins by additional methods. First, we wanted to test the binding of 14F7 to keratinocytes in fresh human tissue. Keratinocytes were collected from fresh healthy skin tissue obtained from patients undergoing breast reduction surgery. Dissociated tissue was processed into single-cell suspensions, which were analyzed by flow cytometry after staining with the 14F7 antibody (**Figure 2C**). Flow cytometry analysis of fresh unfixed skin showed a significant number of keratinocytes positive for 14F7 staining (**Figure 2D** and **Supplementary Figure 2A**).

The presence of Neu5Gc-GM3 ceramides in human keratinocytes was supported by mass spectrometry using HILIC-UPLC FLR/MS. EGCase I is a glycosphingolipid-specific enzyme that allows the specific analysis of ceramide-associated oligosaccharides. **Figure 2E** shows the analysis of a chemically synthesized Neu5Gc-GM3 standard in comparison with the surface ceramide-associated oligosaccharides of keratinocytes. The retention times in the fluorescence spectrum and masses observed in the analysis of human keratinocytes are identical to the ones generated from standards and support the presence of Neu5Gc-containing glycolipids in keratinocytes.

These results show that Neu5Gc is present in human healthy skin.

## Discussion

Changes in sialic acid uptake and sialylation of cancer-associated glycans have been described since many years (1, 8, 29, 30). In particular, upregulation of glycans containing sialic acid (hypersialylation) and incorporation of the non-human sialic acid Neu5Gc have been identified as the most common changes (4). Here, we describe the presence of the Neu5Gc-containing glycolipids in the skin of healthy humans, which is surprising since humans lack the hydroxylase that is responsible for the generation of the glycolyl group (15). However, it has been reported that Neu5Gc can be taken up by the consumption of different mammalian food sources and incorporated into human glycans (9, 10). This xeno-antigen also leads to antibodies that recognize this epitope (31).

Previous analyses have not discovered increased amounts of Neu5Gc in human skin (32), probably because general antibodies against Neu5Gc were used for the detection. Here, we used the specific antibody 14F7 for the detection of Neu5Gc-containing gangliosides by immunostaining. In addition, we confirmed the binding of the 14F7 antibody to formalin-fixed keratinocytes as well as to unfixed keratinocytes from fresh human skin. In addition, we corroborated our finding by an antibody-independent method using mass spectrometry from keratinocytes.

As other healthy tissues have not been found to have a significant positive staining for 14F7, Neu5Gc-GM3 could still be an interesting target, in particular as the main staining was found intracellular in keratinocytes. The 14F7 antibody was tested on cell lines *in vitro* (20, 30, 33), and humanized antibodies of 14F7 have been made and tested in preclinical *in vitro* and *in vivo* models (22). Gangliosides have been targeted for cancer therapy by antibodies and also CAR T cells. For example, GD2 is targeted with chimeric antigen receptor (CAR) T cells and antibody–drug candidates in neuroblastoma and glioma patients (34). However, further tests are needed to determine the potential use of the 14F7 antibody for therapeutic use.

Humans absorb and metabolically incorporate Neu5Gc, enriched in foods of mammalian origin, even while generating xenoreactive, and potentially autoreactive, antibodies against the same molecule (9). Although the detection of Neu5Gc-containing glycolipids in healthy human skin is interesting, it remains unclear how Neu5Gc is taken up by keratinocytes and further studies are needed to determine its exact role and metabolism.

## Data availability statement

The original contributions presented in the study are included in the article/**Supplementary Material**. Further inquiries can be directed to the corresponding author.

## Ethics statement

The studies involving humans were approved by Ethical Committee of Central and Northwestern Switzerland (EKNZ). The studies were conducted in accordance with the local legislation and institutional requirements. The participants provided their written informed consent to participate in this study.

## Author contributions

MM: Conceptualization, Data curation, Formal Analysis, Investigation, Supervision, Writing – review & editing. NR: Formal Analysis, Project administration, Writing – original draft. AZ: Investigation, Writing – review & editing. EK: Resources, Writing – review & editing. A-JB: Data curation, Investigation, Writing – review & editing. JB: Supervision, Writing – review & editing. RF: Conceptualization. AF: Funding acquisition, Supervision, Writing – review & editing. HL: Conceptualization, Funding acquisition, Resources, Supervision, Visualization, Writing – original draft, Writing – review & editing.
