# Supplementary material for: Detection of N-glycolyl-neuraminic acid-containing glycolipids in human skin
Source: Front Immunol. 2023 Nov 29;14:1291292. doi: 10.3389/fimmu.2023.1291292 (PMC10716299; doi:10.3389/fimmu.2023.1291292)
Supplement: Supplementary file 1 [file DataSheet_1.pdf]

Supplementary Material

Detection of N-glycolyl-neuraminic acid-containing glycolipid in human healthy skin

Michela Manni, Natalia Rodrigues Mantuano, Andreas Zingg, Elisabeth Kappos, Anna-Janina Behrens, Jonathan Back, Rainer Follador, Amir Faridmoayer, and Heinz Läubli

Supplementary Methods

ELISA

Synthetic gangliosides Neu5Gc-Gm3 or Neu5Ac-GM3 (Chembind) were dissolved in methanol and coated on a 96-well polysorp plate (NUNC) overnight at room temperature. Next, wells were washed with wash buffer (PBS containing 0.05% Tween 20, PBST) before blocking with 2% BSA in PBST. After washing, antibody 14F7 was applied at a starting top concentration of 1.6ug/ml: 8000x) and incubated for 1 hour at room temperature. Detection was performed using an anti-human IgG conjugated to HRP (Jackson ImmunoResearch, Cat# 109-035-088) and revealed using substrate 3,3′,5,5′-tetramethylbenzidine (TMB). Reaction was stopped by adding 2 M sulfuric acid to each well and absorbance measured at 450 nm by Synergy ELISA plate reader (Biotek).

Supplementary Figure 1

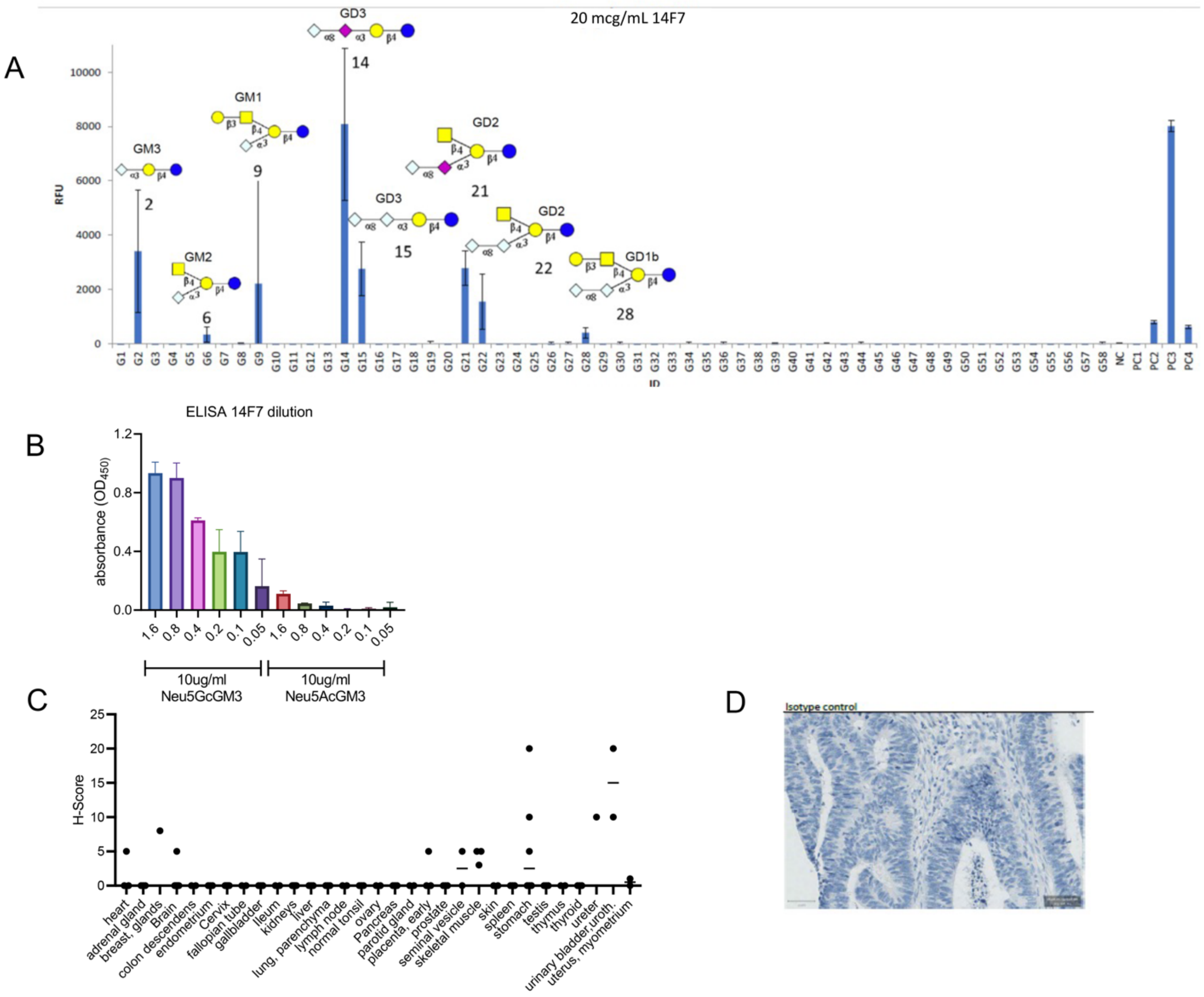

Supplementary Figure 1: **A.** 14F7 specificity for Neu5Gc-GM3 as evaluated by glycan profiling. Graph shows the 14F7 binding to GM3 and other gangliosides. **B.** 14F7 specificity for Neu5Gc-GM3 as evaluated by ELISA. Graph shows the 14F7 binding to Neu5Gc-GM3 or Neu5Ac-GM3. **C.** Neu5Gc-GM3 expression as assessed by 14F7 staining in normal tissues. Graph shows the H-score in the indicated samples. Each dot represents one TMA core. D. Isotype control, secondary antibody only for 14F7 staining in colorectal carcinoma.

Supplementary Figure 2

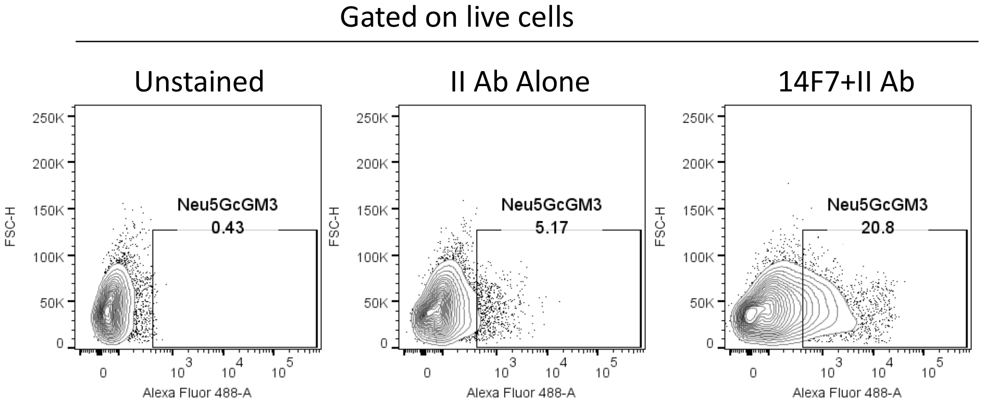

Supplementary Figure 2: Gating strategy used to evaluate Neu5Gc-GM3 expression in fresh human skin by flow cytometry. Unstained control and FMO control (without 14F7 but with secondary antibody) were included .
